# Problematic Gaming and Internet Use but Not Gambling May Be Overrepresented in Sexual Minorities – A Pilot Population Web Survey Study

**DOI:** 10.3389/fpsyg.2018.02184

**Published:** 2018-11-13

**Authors:** Niroshani Broman, Anders Hakansson

**Affiliations:** ^1^Department of Clinical Sciences Lund, Psychiatry, Faculty of Medicine, Lund University, Lund, Sweden; ^2^Malmö Addiction Center, Malmö, Sweden

**Keywords:** gambling disorder, pathological gambling, internet gaming disorder, internet addiction, LGBT, sexual minority, behavioral addiction

## Abstract

**Background:** Substance-related addictive disorders are known to be overrepresented in non-heterosexual individuals, but it is largely unknown whether this is also the case for behavioral addictions such as problem gaming and gambling. This study aimed, in a pilot web survey design, to assess whether problematic gambling, gaming and internet use may be more common in individuals with a non-heterosexual orientation.

**Methods:** An online survey was distributed through media and social media, and answered by 605 individuals (51% women and 11% non-heterosexual). Problem gambling, problem gaming and problematic internet use were measured through structured screening instruments (the CLiP, the GAS and the PRIUSS, respectively).

**Results:** Problem gaming and problematic internet use were significantly more prevalent in non-heterosexual subjects. Instead, problem gambling did not differ between heterosexual and non-heterosexual respondents. Psychological distress and social media use for more than 3 h daily were significantly more common in non-heterosexual respondents. In the overall sample, gaming and gambling were associated statistically.

**Conclusion:** Based on the present pilot online survey, problematic gaming and internet use, but not problem gambling, may be more common in non-heterosexual populations. This area merits more and larger studies, and potentially preventive efforts aimed for non-heterosexual individuals in the population. Possible explanations and study limitations are discussed in the paper.

## Introduction

Gambling disorder, listed as one of the addictive disorders in the DSM-5 manual (American Psychiatric Association, 2013), is believed to have a prevalence of around 0.5% (Abbott et al., 2014), and past-year prevalence of any problem gambling ranges between 0.1 and 5.8% globally (Calado and Griffiths, 2016). Some of the associated risk factors of problem gambling are male sex, age under 30 years and a lower degree of education (Wong et al., 2003; Abbott et al., 2014; Dowling et al., 2017). Meanwhile, digital games (not primarily involving money), despite potentially positive features such as improved short-term memory (Adachi and Willoughby, 2013), have been suggested to be associated with problematic development, similar to a substance dependence (Lemmens et al., 2009; American Psychiatric Association, 2013; Thoresen Wittek et al., 2016), with a diagnostic prevalence of 1–3% among adolescents in European countries (Lemmens et al., 2009; Muller et al., 2015; Rehbein et al., 2015). In the DSM-5 (American Psychiatric Association, 2013), internet gaming disorder is described as a tentative diagnosis, whereas the World Health Organization will include excessive gaming as a disorder in its updated diagnostic manual (ICD-11) (World Health Organization, 2018). Excessive gaming involves loss of control, conflicts with family members and withdrawal symptoms, related to sudden stops in gaming activity (Lemmens et al., 2009). Also, the rise in online gambling among treatment-seeking out-patients with gambling disorder (Håkansson et al., 2017) gives reason to discuss whether there is a connection between gambling with money and an excessive gaming, although the understanding of this association may be incomplete in the scientific discourse (Delfabbro et al., 2009).

In sexual minorities, i.e., individuals with a sexual orientation other than heterosexual, research related to gambling and gaming is scarce. In contrast, an overrepresentation of alcohol and illicit drug use disorders is well documented (Cochran et al., 2004; Corliss et al., 2006; The Swedish Public Health Institute, 2014; National Board of Health and Welfare, 2016). Especially, bi- and homosexual women seem to have a greater relative risk for developing alcohol dependence (Coulter et al., 2018), and it is also reflected in a Swedish register study among same-sex couples (National Board of Health and Welfare, 2016). Research suggests that minority stress within the sexual minority group is associated with disparities in health between heterosexual and non-heterosexual populations (Blosnich et al., 2015). Perceived discrimination as well as experience of physical and psychological violence is more common among the lesbian, gay, and bisexual population than in the general population, which supports the theory that the disparities in health could be a result of minority stress (The Swedish Public Health Institute, 2014; Blosnich et al., 2015; McKay et al., 2017). However, the newer field of non-substance addictions is very sparsely addressed in sexual minorities. One United States study demonstrated that the prevalence of pathological gambling was higher among homo- and bisexual men in comparison to the heterosexual population (Grant and Potenza, 2006). For gaming, previous literature is lacking, but it has been hypothesized that excessive gaming could be more common in sexual minorities, due to the non-stigmatizing features of the online situation. Problem gaming was studied in a clinical sample of transgender individuals, although without demonstrating an overrepresentation of the internet gaming disorder (Arcelus et al., 2017).

In addition to the overrepresentation of non-heterosexual individuals in other addictive disorders, specific factors call for the study of gambling- and gaming-related problems in people identifying as non-heterosexual. Online games may offer opportunities for gender swapping, ‘avatar’ naming, and experimentation with sexual identification, to an increasing extent compared to the ‘real world’ setting (Blodgett et al., 2007; Kelley, 2012; Devlin and Holohan, 2016). On the other hand, a link between gaming behavior and sexual minority identification is far from intuitive, as video games also have been described to be typically hetero-normative (Shaw, 2009; Krobová et al., 2015), and this dualism may add to the complexity of sexual identification and gaming behavior. Thus, these observations call for a research focus on whether excessive internet use or problem gaming may be overrepresented in non-heterosexual minorities, although associations may be complex, and it is also unclear whether this has relevance for other non-substance addictions such as problem gambling.

Based on this research gap, the aim of this pilot study was to investigate whether the prevalence of problem gambling, problem gaming and problematic internet behavior may differ depending on sexual orientation or gender identity.

## Materials and Methods

Data collection was performed with a web-based questionnaire, designed as a self-selective test distributed online between April and June 2017, where the participants clicked a web-link to access the survey. The survey was spread online and presented as a self-test for problem gaming and problem gambling, aiming to address individuals above 15 years of age. The survey was promoted through online advertising in social media and in Swedish news media (including a brief interview carried out with the first author in a regional radio morning show), and two universities assisted in spreading the survey online among students and staff. In order not to bias the inclusion of individuals with respect to sexual orientation or other socio-demographic or other variables, the test did not specifically address any of these sub-groups.

Participants were asked to define sexual orientation, and could choose between *heterosexual*, *bisexual*, *homosexual*, and *other*. In the analysis, sexual orientation was divided into two categories; *heterosexual* represented one group, while homosexual, bisexual and other represented one group together, categorized as *sexual minority group* in this study. Given the low number of transgender respondents, no specific analysis was carried out for transgender identification as a potential risk factor.

The researchers collaborated with a marketing survey company, which set up the questionnaire electronically and handled the incoming data. IP addresses were blocked when collecting data, such that the survey was completely anonymous. It was mandatory to answer all of the questions to be able to continue to the next page in the survey. One question had the response alternative “do not want to answer” and those answers were handled as missing data and excluded in the analysis. The survey was carried out in Swedish, however, with an option to choose from a range of minority languages (very few respondents chose another language than Swedish). Given the pilot and descriptive nature of the study, no power calculation was carried out, and the study included subjects until the pace of study inclusion slowed down.

Informed consent online was necessary for the survey to open. The study was approved by the regional ethics committee, Lund, Sweden (file number 2017/16).

### Instruments and Measures

All measures in this investigation (Table 1) were self-reported. In the development of the survey, already available structured forms were used for the three problem behaviors addressed in the study (problem gambling, problem gaming and problematic internet use), whereas for other variables, simple descriptive questions were used. Questions about age, occupational status, and social media use were mandatory, as well as the three problem behaviors, whereas questions about gender and sexual orientation were optional and could be skipped for subjects unwilling to answer these questions. The same was applied for one question addressing whether the individual had ever felt the need to seek treatment because of mental health problems (yes, no, prefer not want to answer), and a question about how many hours per day they communicate through social media, online chatting (including in-game chat rooms), WhatsApp, Skype or similar services (less than 1, 1–2, 2–3, 3–4, or more than 4 h), and whether the individual had a sufficient amount of people to spend time with, too many people to spend time with, or feelings of loneliness and a wish to have more people to spend time with (aiming to measure social isolation). Altogether, numbers of missing data were low (Table 1). In addition to the questions above, the survey included three previously established and validated questionnaires, i.e., the respective measures of potentially addictive behavior related to gambling for money, video gaming, and internet use. These instruments were the following:

1. The NODS-CLiP (loss of Control, Lying and Preoccupation), validated for the screening of problem gambling, indicated by one or more affirmative answers (Toce-Gerstein et al., 2009).
2. The Gaming Addiction Scale (GAS), validated in a Dutch population (Lemmens et al., 2009), for the assessment of gaming habits, and consists of seven questions, rated on a 5-point Likert scale (never, rarely, sometimes, often, and very often).
3. The Problematic and Risky Internet Use Screening Scale 3 (PRIUSS) (Moreno et al., 2016), consists of three questions, rated on a 5-point Likert scale (never, rarely, sometimes, often, and very often).

**Table 1:** Sample characteristics (*n* = 605).

	% (n) for categorical variables, median (IQR) for continuous variables
Occupational status	
- Employed	59% (*n* = 356)
- Student	34% (*n* = 203)
- Retired	4% (*n* = 27)
- Job-seeking	2% (*n* = 11)
- Other	1% (*n* = 8)
Age	
- 15–18 years	9% (*n* = 57)
- 19–24 years	22% (*n* = 133)
- 25–29 years	11% (*n* = 69)
- 30–39 years	23% (*n* = 138)
- 40–49 years	16% (*n* = 94)
- 50–59 years	11% (*n* = 64)
- 60 years or above	8% (*n* = 50)
Social media use daily	
- Less than 1 h	44% (*n* = 266)
- 1–2 h	23% (*n* = 138)
- 2–3 h	14% (*n* = 85)
- 3–4 h	8% (*n* = 48)
- More than 4 h	11% (*n* = 68)
Gender	
- Female	52% (*n* = 312)
- Male	45% (n = 272)
- Transgender	1% (*n* = 6)
- Missing	2% (*n* = 15)
Sexual identity	
- Heterosexual	90% (*n* = 543)
- Homosexual	3% (*n* = 17)
- Bisexual	6% (*n* = 35)
- Other	1% (*n* = 8)
- Missing	0% (*n* = 2)
Gambling (CLiP) above cut-off	11% (*n* = 64)
Gaming (GAS value)	Median 10 (IQR 7-14)
Gambling (GAS) above cut-off	14% (*n* = 86)
Problem internet use (PRIUSS value)	Median 3 (IQR 0-12)
Number of social contacts outside the internet	
- Would have wished for more, feeling lonely	20% (*n* = 122)
- Satisfactory	71% (*n* = 430)
- Too many	8% (*n* = 47)
- Missing	1% (*n* = 6)
Ever felt need to seek professional help for psychological distress	
- Yes	43% (*n* = 260)
- No	51% (*n* = 310)
- Prefer not to answer	5% (*n* = 28)
- Missing	1% (*n* = 7)

At the end of the survey, a recommendation was provided to the respondents; risk behavior (and an additional message for higher risk) was communicated, along with a recommendation to seek help, and with a stronger emphasis for participants reaching the higher problem level. Cut-offs for this risk assessment were the scoring of one (three) CLiP items, at least ‘sometimes’ on four (all seven) GAS items, or six (nine) or more on the PRIUSS.

### Statistical Methods

All statistical analyses were performed in SPSS version 24. Statistical associations were calculated between problematic gambling, problematic gaming, internet behavior, sexual orientation, number of hours communicating with others online, experience of social contacts outside the internet and experienced need for seeking health care because of psychological distress. Mann Whitney *U*-test was used calculating continuous data and Chi-Square test for categorical data.

## Results

### Sample Characteristics

Answers from 943 participants were collected in the online inquiry, 605 of which were fulfilled and further analyzed. The median age was in the range 30–39 years. Most participants were employed (59%, *n* = 356), 34% (*n* = 203) were studying, 4% (*n* = 27) were retired and 2% (*n* = 11) were job seeking. Heterosexual was the most common sexual orientation (90%, *n* = 543) and 10% (*n* = 62) defined as homosexual, bisexual or other. There were 45% (*n* = 272) defining as men, 52% (*n* = 312) defining themselves as women and 1% (*n* = 6) defining as transgender [gender was missing in two percent (*n* = 15)].

### Gambling, Gaming, Internet Habits, and Sexual Orientation

Problem gambling was not more common in non-heterosexual respondents than in heterosexual (10 vs. 11 percent, *x*^2^ = 0.04, df = 1, *p* = 0.85). In contrast, the median GAS value was significantly higher in the non-heterosexual group (13.0 [IQR 8-17.25]), than in the heterosexual group (9.0 [IQR 7-14]) and the difference between the groups reached statistical significance (*p* < 0.001). Reaching the GAS threshold for problem gaming was more common in non-heterosexual (26 percent, *n* = 16) than in heterosexual individuals (13 percent, *n* = 70, *x*^2^ = 7.61, df = 1, *p* < 0.01). The median value in PRIUSS 3 was 3.0 (IQR 1-5) in the heterosexual group and 4.0 (IQR 2-7) in the non-heterosexual group (*p* = 0.02). Also, social media use for more than 3 h daily was more common in non-heterosexual (32 percent, *n* = 20) than in heterosexual respondents (18 percent, *n* = 96, *x*^2^ = 7.63, df = 1, *p* < 0.01). The experienced need for seeking health care because of psychological distress was 61% (*n* = 38) in the non-heterosexual group, significantly higher than in the heterosexual group where the corresponding percentage was 41% (*n* = 222, *x*^2^ = 9.46, df = 1, *p* < 0.01), whereas social isolation, measured as a too low number of social contacts outside the internet, was not more common in non-heterosexual respondents (19 percent, *n* = 12) than in heterosexual individuals (20 percent, *n* = 110, *x*^2^ = 0.04, df = 1, *p* = 0.83). GAS was significantly higher in problems gamblers (*p* < 0.001), whereas the PRIUSS score was not associated with problem gambling (*p* = 0.91).

## Discussion

The present pilot study described differences in non-substance addictive behaviors, between heterosexual and non-heterosexual groups. The findings in this study suggested that problem gaming and excessive internet use may be more common among lesbian, gay and bisexual individuals than in the heterosexual population, while problematic gambling for money was not associated to sexual orientation. Additionally, the result confirmed a significantly higher level of psychological distress in lesbian, gay and bisexual individuals than heterosexual groups, and a significant correlation between gaming and gambling, in the whole group of participants.

The excessive use of internet and digital games among sexual minorities, i.e., non-heterosexual individuals, compared to the heterosexual group in the present study, could be a further acknowledgment of the vulnerability for addiction-related behaviors and psychological distress within the group. The overrepresentation of alcohol dependence and illicit drug use within sexual minority groups is well known in an American and European context (Cochran et al., 2004; Corliss et al., 2006; National Board of Health and Welfare, 2016). However, no statistical differences were found in problem gambling between the groups in this study, in contrast to Grant and Potenza (2006) study showing that pathological gambling was overrepresented among homosexual and bisexual men. While very sparsely studied previously, the findings of the present study indicate that problematic gaming and internet behavior may need further focus in research and preventive work in sexual minority groups. Interestingly, an increased screening for these two constructs was seen, as well as an increased amount of interactive internet use in non-heterosexual respondents, although this was not explained by a higher degree of social isolation. Hussein and Griffiths (Hussein and Griffiths, 2008) showed that gender swapping in digital games occurred in the majority (57%) of the participants in their study, without revealing the gender that the players normally identified with in the offline world. Considering the stigmatization that could encounter people who are not living hetero-normatively (Blosnich et al., 2015; McKay et al., 2017), the anonymity offered in digital platforms might be experienced as an appealing environment, in contrast to non-digital environments.

Internet gaming disorder has been described to cause significant problems – similar to other addictive behaviors – among adolescents and young adults (Lemmens et al., 2009; Muller et al., 2015; Rehbein et al., 2015). Considering the exposure for psychiatric and substance-related problems among sexual minorities (Cochran et al., 2004; Corliss et al., 2006; National Board of Health and Welfare, 2016), it should be of importance to pay attention to and further investigate non-substance-related addictions within the group. More research is needed, including both quantitative studies in larger samples and qualitative designs, in order to replicate the present pilot study results, and to further clarify possible mediators of this association. For example, while substance-related addictive disorders have been shown to be overrepresented in non-heterosexual populations, the indication seen here of a link to problem gaming and problem internet use does not fully explain the pathways through which this association may appear. In addition to the arguments above, about gender- or identity-specific reasons for online gaming, it cannot be fully established whether this makes non-heterosexual individuals more prone to experience gaming problems simply due to a greater likelihood of gaming and quantitatively more extensive gaming habits, or whether they may be more sensitive to the addictive nature of video games. The findings of the present pilot study may add to previous observations that video gaming in individuals with a non-majority sexual identification may offer a setting where the lived sexual orientation or gender identity can be made possible (Blodgett et al., 2007; Kelley, 2012; Devlin and Holohan, 2016), and that potentially, this may extrapolate into an increase prevalence of gaming-related problems. However, as described above, the link between non-heterosexual identity and video game behavior is likely to be highly complex, as the video game content also has been described to be hetero-normative and even to display negative attitudes toward non-heterosexual identity (Shaw, 2009; Krobová et al., 2015). Thus, more research needs to address in-depth issues above how these different aspects interact in the gaming behavior and potential problem gaming behavior of non-heterosexual individuals.

Also, in settings where problem gaming is screened for or treated, clinicians may need to be aware of a potentially higher prevalence of gaming and internet-related problems in groups with non-heterosexual identification. Although internet gaming disorder is emerging as a disorder in updated diagnostic manuals, clinical treatment and research on therapeutic methods for this condition is hitherto sparse. Thus, in many settings there is not readily a treatment modality where the knowledge about a potential link to sexual orientation can be implemented, and likely screening and prevention efforts are also few. However, the present pilot data and future studies in the area should inform methods for early detection and prevention of a problematic video game behavior.

An incidental finding was the correlation between problematic gambling and gaming (without money). There are still few studies in the area, and it has been described that risk factors of these two constructs are only partially overlapping (Mallorquí-Bagué et al., 2017). While somewhat beyond the scope of the present study, the potential association between problematic gambling and gaming merits further research in larger study samples.

The present pilot study has limitations. This study had a self-selective study design, which may attract mainly participants with a certain interest in gambling, gaming and internet use, and participants in our study were mainly younger adults. Also, the absolute number of non-heterosexual individuals was low, which limits the transferability of the result to sexual minority group. In addition, due to a technical problem, some study items were possible to skip although this was not intended by the authors, such that there were some missing data for a total of 21 respondents, although only for some items. Despite a low number of missing data, a *post hoc* analysis was run for the variable most commonly missing (gender, data missing for 15 cases). This small group of subjects did not differ significantly from individuals with gender data available; neither with respect to problem gambling (*p* = 0.39), problem gaming (*p* = 0.92), PRIUSS value (*p* = 0.80) or the need to seek treatment for psychological distress (*p* = 0.45). Thus, at least from the present data, no systematic differences could be seen with respect to non-substance-related addictive behaviors or psychological distress for the few subjects who did not report gender data.

Also, previous literature has suggested that study questionnaires may demonstrate some invariance in psychometric properties depending on individuals’ sexual orientation. While it is beyond the scope of the present study to further analyze this, it should be borne in mind that our knowledge is limited concerning how instruments addressing gambling, gaming and internet behavior are perceived by individuals from different sexual groups. Likewise, due to a low number of respondents identifying as transgender, the present study does not allow for analyses of other gender identifications; thus, future studies need to include sufficiently large number of respondents for a sub-analysis of transgender individuals to be possible.

In conclusion, this pilot web survey studied potentially addictive behaviors, which have been very sparsely addressed in previous literature, with respect to sexual minority status, and demonstrated that problem gaming and problematic internet use may be more common in non-heterosexual groups. In behavioral addictions, emerging as problematic in recent years, sexual orientation may need to be addressed in prevention and assessment, and more research in the area is warranted.

## Ethics Statement

The study, including its design, information, and consent procedures, was approved by the Regional Ethics Committee Lund, Sweden (file number 2017/16). All participants provided informed consent (which in Sweden can be carried out by the individual from 15 years of age, without need for the consent of a legal guardian).

## Author Contributions

AH was the main responsible of the study. NB was responsible of the practical planning of the study, under AH’s supervision and also wrote the main part of the manuscript. Both authors were carried out the statistical analyses and read and approved the final manuscript.

## Conflict of Interest Statement

The authors declare that the research was conducted in the absence of any commercial or financial relationships that could be construed as a potential conflict of interest. AH receives an overall research funding from Svenska spel AB, the government-owned Swedish gambling monopoly. This funding is non-project-specific (i.e., not aimed specifically for the present project) and does not affect the research idea, study design or interpretations of results of the study.
